# Management of pheochromocytomas and paragangliomas: Review of current diagnosis and treatment options

**DOI:** 10.1002/cam4.6010

**Published:** 2023-05-05

**Authors:** Michal Eid, Jakub Foukal, Dana Sochorová, Štěpán Tuček, Karel Starý, Zdeněk Kala, Jiří Mayer, Radim Němeček, Jan Trna, Lumír Kunovský

**Affiliations:** ^1^ Department of Hematology, Oncology and Internal Medicine, University Hospital Brno Faculty of Medicine, Masaryk University Brno Czech Republic; ^2^ Department of Radiology and Nuclear Medicine, University Hospital Brno Faculty of Medicine, Masaryk University Brno Czech Republic; ^3^ Department of Surgery, University Hospital Brno Faculty of Medicine, Masaryk University Brno Czech Republic; ^4^ Department of Gastroenterology and Internal Medicine, University Hospital Brno Faculty of Medicine, Masaryk University Brno Czech Republic; ^5^ Department of Comprehensive Cancer Care, Masaryk Memorial Cancer Institute Faculty of Medicine, Masaryk University Brno Czech Republic; ^6^ Department of Gastroenterology and Digestive Endoscopy Masaryk Memorial Cancer Institute Brno Czech Republic; ^7^ 2nd Department of Internal Medicine – Gastroenterology and Geriatrics, University Hospital Olomouc Faculty of Medicine and Dentistry, Palacky University Olomouc Olomouc Czech Republic

**Keywords:** biomarkers, chemotherapy, molecular biology, prognostic factor, surgery, target therapy

## Abstract

Pheochromocytomas (PCCs) are rare neuroendocrine tumors derived from the chromaffin cells of the adrenal medulla. When these tumors have an extra‐adrenal location, they are called paragangliomas (PGLs) and arise from sympathetic and parasympathetic ganglia, particularly of the para‐aortic location. Up to 25% of PCCs/PGLs are associated with inherited genetic disorders. The majority of PCCs/PGLs exhibit indolent behavior. However, according to their affiliation to molecular clusters based on underlying genetic aberrations, their tumorigenesis, location, clinical symptomatology, and potential to metastasize are heterogenous. Thus, PCCs/PGLs are often associated with diagnostic difficulties. In recent years, extensive research revealed a broad genetic background and multiple signaling pathways leading to tumor development. Along with this, the diagnostic and therapeutic options were also expanded. In this review, we focus on the current knowledge and recent advancements in the diagnosis and treatment of PCCs/PGLs with respect to the underlying gene alterations while also discussing future perspectives in this field.

## Graphical abstract

Pheochromocytomas are rare neuroendocrine tumors derived from the chromaffin cells of the adrenal medulla. When these tumors have an extra‐adrenal location, they are called paragangliomas and arise from sympathetic and parasympathetic ganglia. According to their affiliation to molecular clusters based on underlying genetic aberrations, their tumorigenesis, location, clinical symptomatology, and potential to metastasize are heterogenous.

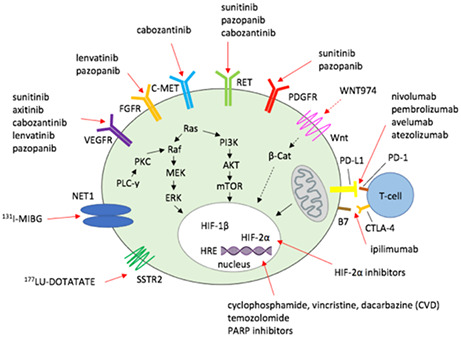

## 1 INTRODUCTION

Incidence of pheochromocytomas (PCCs) and paragangliomas (PGLs) is 0.8/100,000 patients per year.^1^ However, this may be underestimated, as multiple studies have demonstrated that 50% of PCCs/PGLs found at autopsy had neither been clinically detected nor diagnosed.^2^ In certain clinical cases, such as patients with adrenal incidentalomas, multiple endocrine neoplasia type 2 (MEN2), and Takotsubo syndrome, the prevalence of PCCs/PGLs may be much higher.^3^, ^4^, ^5^, ^6^, ^7^ The majority of patients are diagnosed in the fourth and fifth decade of life. Occurrence in childhood is up to 20% of all cases.^8^ Although PCCs/PGLs have a commonly indolent behavior, 15% of PCCs and up to 50% of abdominal PGLs will metastasize.^9^

PCCs are rare tumors originating from the chromaffin cells of the adrenal medulla. PGLs arise from the sympathetic and parasympathetic paraganglia outside of the adrenal gland. These ganglia are most commonly localized in the retroperitoneum, pelvis, thorax, head, and neck, but 85% of PGLs occur below the diaphragm.^10^ The occurrence of PCCs/PGLs is generally sporadic, but one‐fourth of all cases is associated with hereditary syndromes.

Hypoxia‐inducible factors (HIFs) play an important role in the process of tumorigenesis in PCCs/PGLs. These transcription factors regulate energy, iron metabolism, and erythropoiesis. Thus, their dysregulation may lead to cancer development and progression. HIF‐2α is an isoform, which is commonly expressed in multiple tissues—endothelium, kidney, heart, lung, gastrointestinal epithelium, and neural crest cell derivatives, including chromaffin cells. HIF‐2α also regulates processes in the sympathoadrenal lineage, catecholamine synthesis, and secretion. Overexpression of HIF‐2α was found to be associated with metastatic progression and poor prognosis mainly in cancer types derived from the neural crest, including PCCs/PGLs.^11^

## 2 SYMPTOMS AND DIAGNOSTIC LABORATORY

PCCs/PGLs may produce norepinephrine, epinephrine, or dopamine in varying amounts. The clinical manifestation depends on the interaction of norepinephrine or epinephrine with the α‐ and β‐adrenergic receptors in several tissues. Therefore, patients may exhibit variable hormonal manifestations.^12^, ^13^, ^14^ Clinical symptoms related to the paroxysmal or constant secretion of catecholamines may lead to a suspicion of PCCs/PGLs. The most common symptoms are a migraine‐like headache, episodic or sustained arterial hypertension, sweating, palpitations, anxiety, tremor, nausea, weakness, pallor, weight loss, or postural hypotension. The typical triad involving a severe headache, sudden palpitations, and sweating is present in only 24% of patients.^15^ Catecholamine excess may lead to a lethal pheochromocytoma crisis.^16^ In rare cases, PCCs/PGLs produce erythropoietin or adrenocorticotropic hormone and cause polycythemia or Cushing syndrome.^17^, ^18^

However, the occurrence of PCCs/PGLs among patients with sustained arterial hypertension is only 0.05%–0.1%. Adrenal PCCs commonly secrete metanephrines and extra‐adrenal PGLs secrete normetanephrines—the respective O‐methylated metabolites of epinephrine, and norepinephrine.^19^ Interestingly, PGLs in the head and neck location are not usually associated with overproduction of normetanephrines. Nearly, 99% of them are nonfunctioning.^20^ Another case of nonfunctioning PCCs/PGLs may be small lesions that synthesize catecholamines only in small amounts.^21^, ^22^

Catecholamine production can be independent of tumor size and every patient with a suspicion of PCCs/PGLs should be screened for free metanephrines and normetanephrines in plasma or via 24‐h urine collection. As shown in Table 1, PCCs/PGLs belonging to molecular cluster 1 are linked to the noradrenergic phenotype (predominantly increased level of normetanephrine), as cluster 1 type is associated with a deficiency of the enzyme phenylethanolamine N‐methyl transferase that converts noradrenaline to adrenaline.^23^, ^24^ Plasma‐free 3‐methoxytyramine is another useful biomarker produced in PCCs/PGLs with mutations of the succinate dehydrogenase (*SDH*) gene and a deficiency in the enzyme dopamine‐β‐hydroxylase, which is responsible for the catalyzation of catecholamine synthesis (the conversion of dopamine to noradrenaline).^25^, ^26^ In PCCs, up to 20% of patients have normal levels of urine catecholamines due to an intermittent or insignificant secretion in some tumors.^27^ Currently, plasma tests are considered superior to urine analysis.^21^ Elevated levels of metanephrines, normetanephrines, and 3‐methoxytyramine that exceed three times the upper normal limit are considered diagnostic.^19^

**TABLE 1:** Molecular clusters—distribution of PCCs/PGLs and their characteristics.

	Cluster 1A	Cluster 1B	Cluster 2	Cluster 3
Proportion	10%–15%	15%–20%	50%–60%	5%–10%
Signaling pathways	Pseudohypoxic Krebs cycle‐related genes, HIF2α stabilization, angiogenesis	Pseudohypoxia *VHL/EPAS1*‐related genes	PI3K/AKT, RAS/RAF/ERK, mTORC1/p70S6K signaling pathway	Wnt/β‐catenin pathway
Percentage of germline mutations	100%	25%	20%	0%
Gene mutation—germline	*SDHx* (*SDHA*, *B*, *C*, *D*), *SDHAF2*, *FH*, *MDH2*, *GOT2*, *SLC25A11*, *DLST*, *IDH1 HIF2*, *KIF1B*, *HRAS*, *HIF1*	*VHL*, *EGLN1/2*	*RET*, *NF1*, *MAX*, *TMEM127*	‐
Gene mutation—somatic	‐	*VHL*, *HIF2A/EPAS1*	*RET*, *NF1*, *MAX*, *HRAS*	*MAML3*, *CSDE1*
Location	Mostly extra‐adrenal	Adrenal and extra‐adrenal	Adrenal	Mostly adrenal
Age of presentation	Early (≤30 years)	Early, sometimes in childhood	Late	Unknown
Biomarkers	Normetanephrine, 3‐methoxytyramine	Normetanephrine	Mostly metanephrine	High chromogranin A expression, catecholamine profile unknown
Metastatic potential	High–Intermediate	Intermediate–Low	Low	Intermediate

Abbreviations: PCC, pheochromocytoma; PGL, paraganglioma.

At least 3 days prior to the assessment of normetanephrines and 3‐methoxytyramine in plasma and urine, it is necessary to avoid caffeine, black tea, nicotine, alcohol, bananas, cheese, almonds, nuts, chocolate, eggs, and vanilla.^26^

The clinical sensitivity of chromogranin A among patients with PCCs/PGLs is 90%, and this biomarker may serve as a complement to metanephrine assays in a diagnostic setting.^28^ NETest is a standardized liquid biopsy used in neuroendocrine tumor diagnosis. Expression of 51 related genes is analyzed by the use of a real‐time polymerase chain reaction. This recently developed test is superior to chromogranin A.^29^ Pęczkowska et al. demonstrated its diagnostic value also in PCCs/PGLs.^30^

## 3 IMAGING

Chest, abdominal, and pelvic multiphasic computed tomography (CT) or magnetic resonance imaging (MRI) scans are considered standard diagnostic procedures. Functional imaging is more specific and can be used for predicting radionuclide therapy. Several radiopharmaceuticals for single‐photon emission computed tomography (SPECT) and positron emission tomography (PET) are available for the diagnosis, staging, and follow‐up of PCCs/PGLs.

When tumors appear on a meta‐iodobenzylguanidine (MIBG) scan, treatment with high‐specific activity (HSA) ^131^I‐MIBG can be indicated.^31^ MIBG contains a benzyl and a guanidine group and is characterized by the iodination of the benzene ring on the meta position. This iodination makes MIBG a very stable molecule resistant to in vivo metabolism.^32^ MIBG serves as a substrate for the norepinephrine transporter 1 (NET1) in the tumor cell membrane.^33^, ^34^ Thus, this transporter is a target for MIBG absorption into the cell. MIBG can be labeled as ^123^I or ^131^I. Both radioisotopes emit gamma radiation detectable by a gamma camera. ^123^I has a shorter half‐life and is thus the preferred radioisotope for the diagnostic procedure; usually at a dose of 185 MBq. In a therapeutic setting, a high dose level of ^131^I‐MIBG may emit enough beta and gamma radiation which can lead to lethal damage to tumor cells. In this case, the therapeutic dose may reach 37 GBq.^32^ For head and neck PGLs, several studies identified ^111^In‐pentetreotide scintigraphy as being superior to ^123^I‐MIBG scintigraphy.^35^

The diagnostic performance of imaging with single‐photon agents (^123^I‐MIBG, ^111^In‐pentetreotide or ^99m^Tc‐hydrazinonicotinyl‐Tyr3‐octreotide) has been shown to be inferior to imaging with PET radiopharmaceuticals.^36^, ^37^ The PET imaging can be performed with a somatostatin analog (^68^Ga‐DOTA‐SSA), a dopamine and catecholamines precursor (^18^F‐FDOPA), a glucose analog (^18^F‐FDG), or with a norepinephrine analog (^11^C‐HED).^38^ ^68^Ga‐DOTA‐SSA has shown excellent results among patients with PCCs/PGLs. In a systematic review and meta‐analysis, ^68^Ga‐DOTA‐SSA PET/CT showed a significantly higher detection rate (93%) than ^18^F‐FDOPA PET/CT (80%), ^18^F‐FDG PET/CT (74%), or ^123/131^I‐MIBG scan (38%).^39^ This meta‐analysis was compromised by the inclusion of PGLs of various origins and a small number of PCCs.

At present, ^68^Ga‐DOTA‐SSA PET/CT is the preferred imaging modality for extra‐adrenal nonmetastatic PGLs and is becoming the imaging modality of choice for metastatic PCCs/PGLs regardless of genetic background. ^18^F‐FDOPA PET/CT is more effective than ^68^Ga‐DOTA‐SSA PET/CT in the detection of PCCs. So, for confirmation and diagnosis of sporadic PCCs, ^18^F‐FDOPA PET/CT, or ^123^I‐MIBG may be used. ^18^F‐FDOPA can also be used as a second choice alternative to ^68^Ga‐DOTA for sporadic head and neck PGLs. In tumors with mutations in the genes encoding succinate dehydrogenase (*SDHx*—denoting any one of *SDHA*, *SDHB*, *SDHC*, or *SDHD*), ^18^F‐FDOPA together with ^18^F‐FDG PET/CT may be used as a second choice alternative to ^68^Ga‐DOTA‐SSA PET/CT. ^18^F‐FDG PET/CT has a low specificity but shows a strong diagnostic potential for metastatic PCCs/PGLs, particularly those associated with *SDHB* mutations.^36^ Among patients with suspected PCCs/PGLs based on clinical, biochemical, or radiological findings, ^11^C‐HED PET/CT demonstrated high sensitivity (96%), specificity (99%), and positive (96%) and negative (99%) predictive values in the diagnostic workup. However, its routine clinical use is limited because of the complex radiopharmaceutical synthesis and short half‐life of ^11^C.^38^, ^40^

## 4 GENETIC BACKGROUND

Genetic consultation should be provided, especially in patients ≤45 years with multiple or bilateral lesions. About 25% of PCCs/PGLs are associated with inherited genetic disorders. Germline mutations in genes related to the Krebs cycle (*GOT2*, *SLC25A11*, *DLST*, *FH*, *MDH2*, *IDH1*, *SDHA*, *SDHB*, *SDHC*, *SDHD4*), hypoxia‐sensing pathways (*VHL*, *EPAS1*), kinase‐signaling pathways (*MET*, *MERTK*, *RET*, *HRAS*, *NF1*, *MAX*, *TMEM127*), and DNA methylation (*H3F3A*, *DNMT3A*, *KIF1Bb*) have all been associated with susceptibility to PCCs/PGLs.^41^, ^42^, ^43^ Notably, the *SDHx* cluster of genes is associated with high penetrance, early age of onset, intermediate to high risk of metastases, extra‐adrenal location, and poor prognosis.^26^, ^44^

PCCs may be also detected in combination with medullary thyroid carcinoma and hyperparathyroidism within MEN2 syndrome. It is usually caused by a germline mutation in the *RET* proto‐oncogene and is inherited in an autosomal dominant pattern.^45^ In MEN2 syndrome, treatment of PCC should be preferred initially to decrease the risk of a catecholamine storm during further therapeutic interventions.

Germline mutation in *NF1* and *VHL* genes predisposes to PCCs in other inherited tumor syndromes such as neurofibromatosis and Von Hippel Lindau syndrome.^46^

In addition to germline mutations, 40% of cases may harbor a somatic mutation in one of the PCCs/PGLs susceptibility genes such as *VHL*, *RET*, *EPAS1*, *SDHB*, *NF1*, or in genes involved in tumorigenesis such as *HRAS*, *TP53*, *CDKN2A*, and *FGFR1*.^47^, ^48^, ^49^

PCCs/PGLs are therefore determined by a specific driver mutation in up to 70% of cases. This genetic heterogeneity raises significant research interest, aiming to improve prognostic stratification, development of precision therapy, and individualization of treatment approaches. According to the underlying driver mutation, PCCs/PGLs may be divided into three different molecular clusters reflecting their different genetic backgrounds, mechanism of tumorigenesis, and biochemical and clinical features.^50^

In cluster 1‐related tumors, germline and somatic mutations activate pathways that mimic hypoxia signaling. Cluster 1 mutations are divided into two subgroups:

- cluster 1A—pseudohypoxic Krebs cycle‐related PCCs/PGLs that are associated with mutations in the following genes: *SDHx*, *SDHAF2*, *FH*, *MDH2*, *GOT2*, *SLC25A11*, *DLST*, and *IDH1*.
- cluster 1B—pseudohypoxia *VHL/ESPAS1*‐related PCCs/PGLs in which mutations in the following genes are present: *VHL*, *HIF2A/EPAS1*, and *EGLN1/2*.

It is worth noting that about 60% of metastatic PCCs/PGLs are associated with cluster 1 mutations (the highest risk for mutations in *SDHB* and *SDHA*, the lowest in *SDHC*). Interestingly, among the cluster 1A/B‐related PCCs/PGLs, there are some differences in primary tumor location.^51^

In cluster 2, mutations in the *RET* proto‐oncogene, *NF1* tumor suppressor, *HRAS*, *MAX*, and *TMEM127* may lead to overactivation of the PI3K/AKT, mTORC1/p70S6K, and RAS/RAF/ERK signaling pathway with subsequent induction of cancerogenesis, tumor cell proliferation, survival, and deregulation of metabolism (switch to glycolysis and glutaminolysis).^52^, ^53^, ^54^ The majority of mutations lead to an adrenergic phenotype with high levels of metanephrines (*MAX*‐related PCCs secrete normetanephrine). These PCCs usually have a better prognosis and low metastatic potential (~4%).^51^

In cluster 3, *MAML3* fusion gene and *CSDE1* somatic mutations affect and overactivate the Wnt/β‐catenin pathway, which is responsible for the regulation of metabolism, angiogenesis, proliferation, and invasion.^55^ Cluster 3‐related PCCs may be detected and followed up by the chromogranin A level.^52^ These tumors are usually associated with a poor prognosis due to the high risk of recurrence and metastases. A summary of the cluster features is listed in Table 1.^26^, ^41^, ^52^

## 5 CRITERIA FOR MALIGNANCY

Despite the fact that most PCCs/PGLs are commonly indolent diseases with nonaggressive behavior, all PCCs/PGLs have a metastatic potential as seen in Table 1. Up to 20% of patients present with de novo metastatic diseases, particularly those with mutation in *SDHA* and *SDHB*.^56^, ^57^

In daily practice, it is difficult to distinguish between benign and aggressive PCCs/PGLs with metastatic potential as the histological features tend to be identical with only minute differences. Similarly, molecular and biochemical markers for metastasis prediction are still limited. Risk stratification systems such as Pheochromocytoma of the Adrenal gland Scaled Score (PASS) and Grading system for Adrenal Pheochromocytoma and Paraganglioma (GAPP) do not reflect the tumor mutational profile, and have an insufficient predictive ability.^26^ The PASS scoring system was originally designed for PCCs and includes only histologic risk factors (capsular or vascular invasion, mitotic rate, and necrosis). However, the meta‐analysis from Stenman et al. revealed that a PASS score of <4 is highly indicative of benign behavior for both PCCs and PGLs. Regarding the prediction of malignant behavior, the positive predictive value of this score is low. These findings indicate that the PASS scoring system should be only used to rule out the malignant potential of PCCs/PGLs.^58^ The GAPP algorithm involves biochemical (type of catecholamine production), and histopathologic features (KI‐67 proliferation index) combined with clinical data to assess both PCC and PGL. Higher values are associated with the aggressiveness of the disease.^59^, ^60^, ^61^ The GAPP score also demonstrates a strong rule‐out function due to its high negative predictive value.^58^, ^62^ Thus, those aggressive PCCs/PGLs with high metastatic potential are recognized only when lymph node infiltration or distant metastases are detected.^63^ PCCs/PGLs mainly metastasize to the lymph nodes, bones, liver, and lungs. Extra‐adrenal PGLs can spread aggressively and invade the surrounding structures.^64^

However, Einsenhofer et al. demonstrated that the plasmatic 3‐methoxytyramine level is 4.7‐fold higher in patients with metastases compared to those without. This biomarker is now considered to be the most accurate discrimination test between localized and metastatic disease.^56^ Furthermore, Patterson et al. studied the role of tissue noncoding microRNA (miR) and revealed that miR‐483‐5p, miR‐101, and miR‐183 were significantly overexpressed in malignant PCCs compared with their benign variants.^65^ In addition, multiple other biomarkers were analyzed as potential predictive factors of malignancy. The main focus was on clinical features (e.g., tumor size and location), biochemical markers (e.g., catecholamines, dopamine, chromogranin A, and neuron‐specific enolase), histopathology (e.g., grading and immunohistochemistry), and genetic markers (e.g., somatic and germline gene mutations, association with clusters 1–3, and DNA methylation). Nevertheless, the prediction role of the majority of these biomarkers is still limited due to the insufficient number of analyzed samples, the minority of metastatic cases, and the differences in treatment approaches.^66^

## 6 CURRENT TREATMENT RECOMMENDATION FOR RESECTABLE TUMORS

Despite advancements in systemic treatment, adrenalectomy remains the only curative treatment for locoregional disease. Preoperative administration of α‐adrenergic receptor blockers decreased the postoperative mortality from 40% to 1%–3%.^67^, ^68^, ^69^ At least 10–14 days prior to surgery, every patient should start with this prevention treatment, including volume repletion with a low‐salt diet. Phenoxybenzamine 10 mg BD or TDS has been a conventional drug used in this indication. Other options include terazosin, doxazosin, and prazosin. During surgery, intravenous administration of the nonselective alpha blocker phentolamine can also be considered.^70^, ^71^ In selected cases, calcium‐channel blockers and β‐adrenergic receptor blockers may be added preoperatively to minimize intraoperative hemodynamic instability and related adverse outcomes.^72^, ^73^

The surgery can be performed as an open procedure (anterior transabdominal, posterior, and flank approach) or as a minimally invasive technique (laparoscopic or robotic). The main aims are to avoid catecholamine surges due to mechanical pressure on the tumor during dissection, disruption of the adrenal capsule, and tumor cells dissemination caused by excessive manipulation. With increasing experience, including the application of the principles of “dissecting the patient from the tumor”, the laparoscopic approach was proven to be have similar outcomes in terms of intraoperative hemodynamic instability compared with open resection and even showed less intraoperative blood loss with faster postoperative recovery.^74^, ^75^ The rates of positive margins in adrenalectomy for malignant PCC were similar compared with the minimally invasive and open approach.^76^

Laparoscopic adrenalectomy can be performed via a transabdominal (lateral transperitoneal) or retroperitoneal approach. The first one enables earlier ligation of the adrenal vein and a wider working space. However, it requires mobilization of the adjacent organs. The retroperitoneal (posterior retroperitoneoscopic) approach does not include entering the abdominal cavity, which is preferred in patients with suspected adhesions due to previous operations. However, obtaining control during the conversion can be more difficult. The previously recommended “vein first technique”, wherein the adrenal vein is ligated prior to further dissection, does not seem to be necessary anymore.^77^, ^78^, ^79^ Data regarding the usage of the robotic approach are limited. The learning curve for robotic adrenalectomy seems to be 20 cases.^80^ Prospective, randomized studies are required to establish the role of robotic surgery. Although the minimally invasive approach appears to be safe even for tumors over 5 cm in size, some authors still recommend open surgery for tumors larger than 5–6 cm.^81^, ^82^, ^83^, ^84^

Partial adrenalectomy is reserved only for patients with hereditary syndromes such as in MEN2 or Von Hippel Lindau, in which bilateral adrenalectomy is avoided to prevent steroid dependency.^85^ Intraoperative ultrasound can optimize tumor localization in both tissue‐sparring techniques as well as standard adrenalectomies.

Surgical resection also remains the main treatment for PGLs. While the preoperative medical preparation in functional PGLs is similar to that of PCCs, the risks related to surgery vary according to the localization. Evidence is represented mostly by case series, with no clear guidelines available. Resection of most parasympathetic head and neck PGLs carries the risk of cranial nerve palsy and vascular injury, especially of the carotid artery. Given the rich blood supply, preoperative chemoembolization was suggested to decrease blood loss and operative time. Although this seems to be an efficient method, there is still no consensus on this matter.^86^ Radiotherapy might be preferred to surgery in selected cases of jugular and jugulotympanic PGLs, especially in larger tumors or in the case of skull involvement.^87^ Thoracic PGLs represent only 1%–2% of all PGLs and can present as an endobronchial hypervascular mass.^88^ Abdominal PGLs are most commonly found along the aorta, especially at the root of the inferior mesenteric artery. Close proximity to large vessels and the renal arteries does not necessarily preclude a minimally invasive approach.^89^ Retroperitoneal PGLs are preoperatively often misdiagnosed for a different histological entity or are falsely considered to be intraabdominal.^90^ Similar to other locations, the blood supply is rich. However, the size can be considerably larger compared with other extra‐adrenal locations. Spinal PGLs occur mostly in the lumbar region and apart from resection itself, laminectomy, or spinal fusion can be required as well.^91^

After resection, adjuvant chemotherapy is not supported by results from clinical trials and is thus not recommended.^26^

Open surgery may be also favored if metastatic disease is suspected and in the case of large vessel invasion or regional nodal disease. While surgical debulking in metastatic PCC seemed to be inefficient due to low rates of biochemical response, more recent studies suggest improved overall survival.^92^, ^93^, ^94^, ^95^ The resection of the primary tumor in the context of metastatic disease can prevent potential local complications resulting from further invasion, decrease cardiovascular‐related mortality, and alleviate symptoms.^84^ Beneficial impact on oncological outcomes has also been suggested, namely by increasing the efficacy of systemic treatment.^96^

## 7 FOLLOW‐UP

Surveillance during the post‐resection period includes regular physical examination, searching for the potential presence of catecholamine‐related symptoms (e.g., high blood pressure, sweating, palpitations, tremor, etc.), and biochemical testing (plasma‐free or 24‐h urine fractionated metanephrines). This should be done every 3–12 months during the first year, every 6–12 months in the first 3 years, and then annually up to 10 years after resection. In cluster 1A/B‐ and 3‐related PCCs/PGLs, where the risk of metastatic dissemination is intermediate to high, an MRI from the base of the skull to the pelvis is recommended every 12 months initially, then every 12–24 months. A chest CT is preferred if lung metastases are suspected. In cluster 2‐related PCCs/PGLs, abdominal and pelvic MRI should be performed at least every 5 years.^51^, ^71^

However, the presence of inherited genetic neuroendocrine disorders may modify this scheme.^71^ Special attention should be given to patients with known risk factors for higher metastatic potential and worse prognosis, such as the presence of germline *SDHA/B* mutations, extra‐adrenal locations, multiplicity, and young age (<20 years).^26^, ^56^, ^97^, ^98^, ^99^, ^100^

## 8 CURRENT TREATMENT RECOMMENDATION FOR NONRESECTABLE TUMORS

Approximately, one‐fifth of all patients have a de novo metastatic disease. For unresectable, nonfunctioning tumors, observation is possible, especially if the patient has no tumor‐related symptoms. In this setting, the progression‐free survival (PFS) rate at 1 year is 50%.^101^

However, in the metastatic setting, multiple treatment options are available. Cytoreductive surgery, where the goal is to decrease symptoms related to advanced disease and excessive production of catecholamines by chromaffin cells, can be considered. Radiotherapy may follow in order to decrease symptoms. Radiotherapy and radiosurgery are also recommended in a palliative care setting where bone metastases have developed or an oligometastatic disease is present and is associated with pain or neurological symptoms.^102^, ^103^ In an oligometastatic setting, minimally invasive procedures such as radiofrequency ablation, cryoablation, and ethanol injection can be recommended on an individual basis.^104^

**FIGURE 1:**
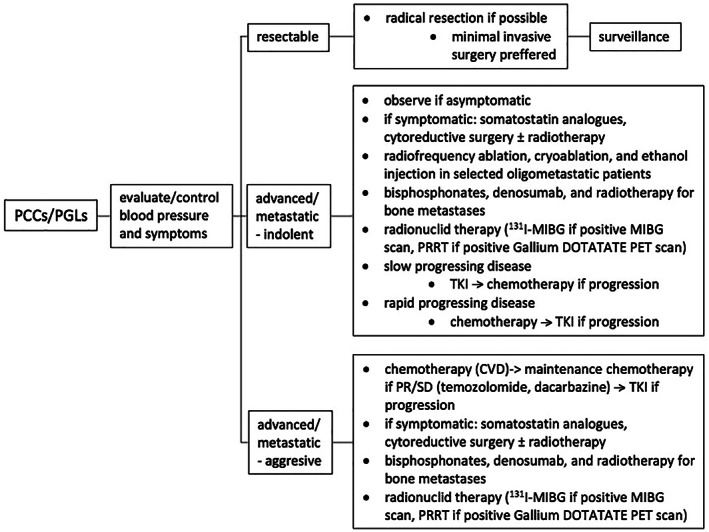
Treatment options in resectable, advanced, and metastatic PCCs/PGLs. PCC, pheochromocytoma; PGL, paraganglioma.

Currently, radionuclide therapy, chemotherapy, and tyrosine‐kinase inhibitors (TKIs) are the standard care in locally advanced/metastatic diseases. Treatment options for all stages of PCCs/PGLs are illustrated in Figure 1.^26^ Surveillance among patients with advanced or metastatic disease who are not candidates for surgery includes regular clinical controls, blood pressure measurement, and biomarkers testing. Besides standard CT restaging, radionuclide imaging such as MIBG SPECT/CT or SSA PET/CT should also be considered prior to radionuclide therapy.^71^

### 8.1 Radionuclide therapy

Uptake of ^131^I‐MIBG is dependent upon the expression of NET1, which serves as a target for theranostic agents.^105^ The efficacy of the ^131^I‐MIBG radionuclide therapy among patients with MIBG‐avid metastatic PCCs/PGLs was demonstrated in multiple trials where complete (CR) or partial responses (PR) were observed in 10% to 34% of patients, whereas the disease control rate (DCR) was observed in up to 92% of treated patients.^62^ Noto et al. demonstrated a clinical benefit of HSA ^131^I‐MIBG in a single‐arm, multicenter, phase I trial. Twenty‐one patients were enrolled, and the median therapeutic dose was 21.13 GBq. All four patients (19%) who reached a PR received >18.5 GBq. Complete or partial biochemical responses for serum chromogranin A and total metanephrines were observed in 80% and 64% of patients, respectively. Overall survival at 2 years was 61.9%.^106^ The single‐arm phase II trial conducted by Pryma et al. enrolled 68 patients with metastatic PCCs/PGLs who received at least one therapeutic dose of HSA ^131^I‐MIBG. The median cumulative ^131^I‐MIBG dose was 35.7 GBq. Seventeen (25%) of them reached a durable reduction of antihypertensive medication. From among 64 evaluable patients, 59 (92%) patients had a PR or a stable disease (SD) as the best response within 12 months. The median overall survival (mOS) was 36.7 months. The most common side effects were nausea, myelosuppression, and fatigue.^107^ When a second application is indicated, at least 90 days of delay should be considered until platelets and neutrophils reach normal limits.^108^ Long‐term side effects are also possible. The most severe are secondary hematological malignancies such as myelodysplastic syndrome, acute myeloid, and acute lymphocytic leukemia.^107^

If tumors are positive on somatostatin receptor (SSTR) imaging, the peptide receptor radionuclide therapy (PRRT) with ^177^Lu‐DOTATATE or treatment with octreotide or lanreotide (in case of symptoms) may be also recommended. There is only limited data available confirming the effect of PRRT in patients with PCCs/PGLs. Preliminary experience suggests low toxicity and favorable efficacy in disease control.^109^ Kong et al. assessed PRRT outcomes among 20 patients with PCCs/PGLs from two referral centers. In conclusion, PRRT can lead to a clinical (reduction of antihypertensive medication, partial response, or stable disease) and a biochemical response (decrease of chromogranin A level). Moreover, PRRT is associated with a low toxicity profile.^110^ Nastos et al. analyzed retrospective data from patients with progressive or metastatic PCCs/PGLs treated either by PRRT (16 patients) or ^131^I‐MIBG (14 patients) therapy. PRRT was associated with significantly increased PFS and response to treatment compared with ^131^I‐MIBG therapy. Toxicity was comparable in both groups. When the subgroup of patients with PGLs was analyzed, OS, PFS, event‐free survival (EFS), and responses to treatment were significantly higher in the PRRT group.^111^

### 8.2 Chemotherapy

Chemotherapy is still a basic option for metastatic disease. It should be considered particularly among tumor subtypes not suitable for radionuclide therapy or in tumors with aggressive growth. Prospective randomized phase II and III trials are not available. Thus, an optimal regimen has not yet been defined. The most studied combination of cytostatics was the Averbuch CVD regimen (cyclophosphamide 750 mg/m^2^ on day 1, vincristine 1.4 mg/m^2^ on day 1, and dacarbazine 600 mg/m^2^ on days 1 and 2, at 21 days intervals). Niemijer et al. analyzed results from four clinical studies concerning a total of 50 patients treated with CVD for metastatic PCCs/PGLs. According to this analysis, the CVD regimen led to a PR or a CR in 46%–55% of patients.^112^, ^113^, ^114^, ^115^ Only one of these studies confirmed a significant survival benefit of CVD chemotherapy. If a response was observed, mOS in these patients was 6.4 years versus 3.7 years for nonresponders. The CVD regimen should be considered mainly in the case of rapid progression within 6 months. The most common side effects associated with CVD were myelosuppression, neuropathy, nausea, vomiting, and hemorrhagic cystitis.^96^

Tumors with a germline mutation in the *SDHB* gene have more aggressive behavior. However, Jawed et al. retrospectively analyzed 12 patients with PCCs/PGLs associated with *SDHB* mutation treated with the CVD regimen. A median of 20.5 cycles (range 4–41) was administered. A dose reduction was necessary for all treated patients. The PR was described in eight patients (67%), while CR was confirmed in the next two patients (17%). The median duration of response was 478 days. Median PFS and OS were 930 and 1190 days, respectively. A prolonged therapy resulted in continued incremental tumor reduction. Thus, the CVD regimen may be more effective in the treatment of patients with metastatic PCCs/PGLs with a mutation in the *SDHB* gene.^116^

Temozolomide is a DNA‐alkylating agent and oral metabolite of dacarbazine. Its efficacy has been already demonstrated in glioblastomas and neuroendocrine tumors of the gastrointestinal tract. In PCCs/PGLs, temozolomide monotherapy is also effective and it may be considered as a single‐agent treatment, or alternative maintenance treatment after 6–9 cycles of CVD chemotherapy if stabilization or regression was reached. Usual schedule is 150 mg/m^2^ on days 1–5, at a 28‐day interval. Metronomic regimen with long‐term and low‐dose temozolomide (75 mg/m^2^ per day, 3 weeks on, 1 week off) is a possible option, which can be considered in case of toxicity during a standard schedule. Hadoux et al. published results from a retrospective analysis of patients with metastatic PCCs/PGLs with *SDHB* mutation treated by temozolomide monotherapy. Mutation of the *SDHB* gene was detected in 10 patients (67%). With a median follow‐up of 35 months, mPFS was 13.3 months. PR was observed only in tumors with *SDHB* mutation and reported in five patients (33%), SD was reported in seven patients (47%), and PD in three patients (20%). The analysis revealed that germline mutation in the *SDHB* gene was associated with hypermethylation of the O‐6‐methylguanine‐DNA‐methyltrasferase promoter and its low expression. In conclusion, this analysis demonstrates the antitumor activity of temozolomide in patients with PCCs/PGLs and similarly, like the CVD regimen, the efficacy of temozolomide is associated with the presence of *SDHB* gene mutation.^117^ Thus, *SDHB* mutation may serve as a predictive marker for the response to chemotherapy. A summary of chemotherapy regimens used in advanced and metastatic PCCs/PGLs can be found in Table 2.

**TABLE 2:** Chemotherapy in advanced and metastatic PCCs/PGLs.

Authors	Methodology	No. of evaluable patients	Treatment regimen	Median no. of treatment cycles	Biochemical responses	Response	Response duration (months)	OS
Huang et al.	Nonrandomized, single arm	18	CVD	18	72%	11% CR, 44% PR	20	mOS 3.3 years (from the on‐study date)
Ayala‐Ramirez et al.	Retrospective	52	CD ±Dx ±V	6.9	NA	25% PR	NA	5 years OS rate 51%
Tanabe et al.	Retrospective	17	CVD	NA	47%	47% MR or PR	40	NA
Hadoux et al.	Retrospective	15	TEM	7	25%	33% PR	13	3 years OS rate 55%

Abbreviations: C, cyclophosphamide; CR, complete response; D, dacarbazine; Dx, doxorubicin; MR, molecular response; NA, not available; OS, overall survival; PCC, pheochromocytoma; PGL, paraganglioma; PR, partial response; V, vincristine; TEM, temozolomide.

### 8.3 Targeted therapy

TKIs target and inhibit tyrosine kinase enzymes responsible for the activation of proteins by signal transduction cascades inside tumor cells, such as Ras/Raf/MEK/ERK, PI3K/AKT/mTOR, and PLC‐γ/PKC pathways.^118^ TKIs also inhibit the tumor microenvironment including angiogenesis and may block tumor growth and spread. Based on results from phase III trials, they were approved for the treatment of thyroid, hepatocellular, kidney, and pancreatic neuroendocrine malignancies. Their antiangiogenic effect plays a crucial role also in the treatment of PCCs/PGLs. Most of the tyrosine kinase receptors were overexpressed in these tumor types, but the expression was not homogeneous.^119^

One of the most investigated TKIs is sunitinib. This oral, small‐molecule multi‐targeted inhibitor of tyrosine kinases blocks the angiogenesis and tumor cell proliferation by targeting multiple receptors, such as the receptors for platelet‐derived growth factors, the receptors for vascular endothelial growth factors (VEGFs) 1 and 2, and RET or KIT receptors. In vitro, sunitinib decreases cell proliferation mostly by targeting the cell cycle, DNA metabolism, and cell organization genes.^119^ Sunitinib is already approved for the treatment of metastatic renal cancer, gastrointestinal stromal tumors, and pancreatic neuroendocrine tumors. O'Kane et al. conducted a single‐arm multicenter phase II trial with sunitinib, in which 25 patients were enrolled between 2009 and 2016. Surgical resection of primary or metastatic sites had been previously performed in 16 patients (64%), and three patients (12%) had been pretreated by chemotherapy. Sunitinib was administered at a starting dose of 50 mg (4 weeks on/2 weeks off). The median dose intensity of sunitinib was 40 mg (25–50 mg). The DCR was 83% and mPFS 13.4 months. Germline mutations in *SDHA*, *SDHB*, and *RET* genes were detected in 3 patients (13%) and were associated with a PR. No CR was observed in this study. The median time on treatment was 12.4 months and grade 3–4 toxicities were manageable, with the most common being fatigue (16%) and thrombocytopenia (16%). The primary endpoint of the study was DCR, which was met. However, the overall response rate (ORR) in the whole study group was low. The greatest benefit was observed among patients with germline mutations in *RET* or *SDHx* genes. The study was closed prematurely due to slow accrual.^120^ Recently, the first randomized, multicenter, placebo‐controlled phase II trial with sunitinib has been initiated in patients with advanced and metastatic PCCs/PGLs (NCT01371202). The primary outcome is PFS at 12 months. Recruitment is completed (78 patients) and final results have yet to be presented.

Cabozantinib is a potent TKI inhibitor that is also under investigation in metastatic settings in phase II trial (NCT02302833). In addition to VEGFRs inhibition, cabozantinib targets the c‐Met receptor. Preliminary results derived from 14 patients have shown ORR of 45% and mPFS of 11.2 months. The side effects of cabozantinib were expectable and similar to those of sunitinib.^121^

Pichun et al. conducted a phase II trial with another TKI axitinib. Only nine patients have been enrolled. The primary endpoint was PFS, secondary endpoints were OS, ORR, and safety. The median number of systemic treatments prior to axitinib was 1 (0–3). Three patients achieved a PR, and the next five patients had mild regressions which did not meet PR criteria. Regression has been associated with reductions in plasmatic catecholamines. Only one progression was observed, and grade 3–4 toxicities included hypertension, fatigue, diarrhea, and mucositis. A reduction of the starting dose of axitinib from 5 to 3 mg and 2 mg was necessary for eight patients (89%). Preliminary data of this trial demonstrates that axitinib may result in tumor shrinkage in patients with malignant PCCs/PGLs.^122^ Axitinib has been under investigation also in an open‐label, nonrandomized phase II trial (NCT01967576). Twelve patients were analyzed and DCR was achieved in 83% of them.

Lenvatinib inhibits VEGFRs and fibroblast growth factor receptor pathways and is already approved for the treatment of patients with advanced thyroid cancer, hepatocellular cancer, and in combination with everolimus as a treatment option for clear cell renal cancer. The efficacy of lenvatinib in patients with PCCs/PGLs was evaluated in a prospective phase II trial (NCT03008369), but this study was terminated due to a slow accrual rate. Nelson et al. retrospectively analyzed patients treated with lenvatinib for advanced PCCs/PGLs. Of the 11 evaluated patients, 8 had measurable disease and the DCR was 63% (5 PR and 3 SD).^123^

Pazopanib was studied in the phase II trial, where only seven patients with advanced PCCs/PGLs were enrolled. Only one patient had a confirmed PR with a duration of 2.4 years. The treatment of the rest of the patients was discontinued due to disease progression (four patients), withdrawal (one patient), and grade 4 cardiomyopathy (one patient). Median PFS and OS were 6.5 and 14.8 months, respectively. The most common side effects were severe arterial hypertension (50%), diarrhea, hematuria, headache, and fatigue.^124^

Everolimus is a mammalian target of rapamycin (mTOR) inhibitor. The efficacy was demonstrated among patients with advanced pancreatic neuroendocrine tumors, renal cancer, and in combination with exemestane in postmenopausal females with hormone‐dependent, HER2‐negative breast cancer. Among patients with PCCs/PGLs, only modest efficacy was demonstrated in the phase II trial.^125^

Belzutifan is a second‐generation, small‐molecule HIF‐2α inhibitor with increased potency and an improved pharmacokinetic profile when compared to other HIF‐2α inhibitors, like PT2385 and PT2399.^126^ Belzutifan has already demonstrated its efficacy among patients with VHL‐associated renal cancer, where ORR was observed in 49% of the patients.^127^ Currently, this new HIF‐2α inhibitor is evaluated in an open‐label, single‐group phase II trial (NCT04924075) among patients with advanced PCCs/PGLs with the primary endpoint being ORR.

**FIGURE 2:**
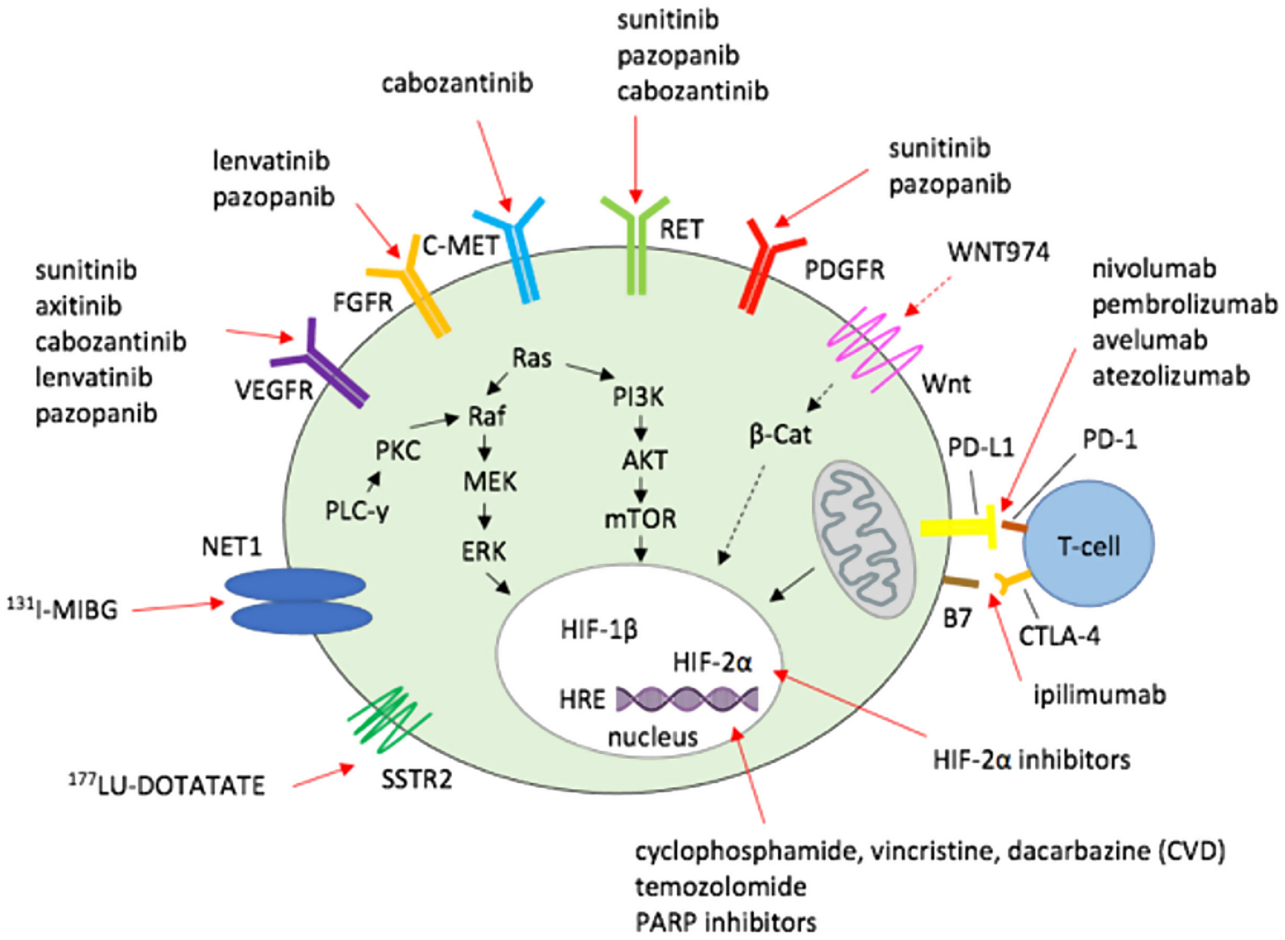
Therapeutic options: TKIs target multiple receptors and inhibit various signaling pathways. Radionuclides enter tumor cells through the specific NET1 receptor. Chemotherapy and PARP inhibitors block cell proliferation and growth at the level of DNA. Immunotherapy activates killer T cells by blocking the binding of PD‐1 and CTLA‐4 receptors with PD‐L1 and B7. HIF‐2α inhibitors block the interaction with a specific‐hypoxia responsive element (HRE) in the proximal region of the tyrosine‐hydroxylase promoter. Inhibition of the Wnt/β‐catenin pathway is promising therapy under investigation. Alterations in mitochondrial metabolism may lead to tumorigenesis. HIF, hypoxia‐inducible factor; TKI, tyrosine‐kinase inhibitor.

There are other multiple potential targeted therapies, which have already demonstrated efficacy or are currently under study. These include poly(ADP‐ribose) polymerase inhibitors targeting the DNA repair system, SSTR2 analogs (octreotide/lanreotide), inhibitors of Wnt/β‐catenin pathway (WNT974, PRI‐724) for cluster 3‐related tumors, and DNA methyltransferase inhibitors (Figure 2).^26^, ^128^, ^129^

### 8.4 Immunotherapy

Immunotherapy has already become a milestone in the treatment of multiple types of cancer. The role of immunotherapy in metastatic PCCs/PGLs was evaluated in several phase I and II trials. Jimenez et al. presented results from a phase II study, where 11 patients with metastatic disease were treated with the anti‐PD1 antibody pembrolizumab. Four patients (36%) exhibited no progress at 27 weeks, which was the primary endpoint. The ORR was 9% and the clinical benefit rate was 73%. The other secondary endpoints were PFS and OS, which were 5.7 and 19 months, respectively. Thus, pembrolizumab in monotherapy has presented only modest antitumor activity, regardless of programmed death‐ligand 1 (PD‐L1) expression or hereditary background. No grade 4–5 adverse events were observed.^130^

Other checkpoint inhibitors are currently under evaluation in multiple clinical trials for advanced PCCs/PGLs. Programmed cell death inhibiting receptor 1 (PD‐1) inhibitor nivolumab in phase I/II trial (NCT04187404), PD‐L1 inhibitors avelumab in phase I trial (NCT02923466) and atezolizumab in phase II trial (NCT04400474). A combination of nivolumab and cytotoxic T‐lymphocyte antigen 4 (CTLA‐4) inhibitor ipilimumab is currently under study in two phase II trials (NCT03333616, NCT02834013).^131^

## 9 CONCLUSION

PCCs/PGLs are rare tumors for which clinical symptoms may be attributed most commonly to cardiovascular comorbidities. Similarly, radiological findings may be falsely considered lymphomas or other tumor types. Correct diagnostic workup should be performed without delay. Biochemical tests such as plasma metanephrines, 3‐methoxytyramine, and chromogranin A are useful and recommended when PCCs/PGLs are suspected. Besides CT and MRI scans, functional imaging, such as ^68^Ga‐DOTA‐SSA PET/CT and ^18^F‐FDOPA PET/CT, is currently also preferred. Recent research has significantly enriched our knowledge about the molecular background and heterogeneity of these malignancies. Currently, we recognize three different molecular clusters according to the underlying driver mutations. These clusters reflect different genetic backgrounds, tumorigenesis, biochemical, and clinical features of PCCs/PGLs. Apart from the prognostic role of these mutations, they may also become potential predictors for precision and target therapy. Genetic counseling should always be considered due to a relatively high association with inherited genetic disorders.

Whenever it is possible, radical surgery is preferred as it is the only curative option. Cluster 1‐ and 3‐related PCCs/PGLs have intermediate to high metastatic potential and there is a higher risk of recurrence. Moreover, about 20% of patients are diagnosed in an advanced stage, which is usually associated with unpleasant clinical symptoms and a deteriorating quality of life. In the palliative setting, standard chemotherapeutic regimens such as CVD or temozolomide are widely used, but they are not supported by results from randomized phase II/III trials. Patients with MIBG‐avid metastatic PCCs/PGLs benefit from HSA ^131^I‐MIBG therapy. Similarly, among patients whose tumors are positive on SSTR imaging, PRRT therapy with ^177^Lu‐DOTATATE should be considered. Along with recent advances in research, multiple new therapeutic approaches such as targeted therapy are under evaluation and the first results are promising.

## AUTHOR CONTRIBUTIONS

**Michal Eid:** Conceptualization (lead); data curation (equal); funding acquisition (lead); resources (lead); writing – original draft (lead). **Jakub Foukal:** Conceptualization (supporting); data curation (equal); formal analysis (supporting); validation (supporting); writing – original draft (supporting); writing – review and editing (supporting). **Dana Sochorová:** Formal analysis (supporting); investigation (equal); resources (supporting); writing – original draft (supporting); writing – review and editing (supporting). **Štěpán Tuček:** Methodology (supporting); supervision (equal); validation (equal); writing – review and editing (equal). **Karel Starý:** Supervision (supporting); writing – original draft (supporting); writing – review and editing (equal). **Zdeněk Kala:** Methodology (supporting); supervision (supporting); writing – review and editing (supporting). **Jiří Mayer:** Funding acquisition (supporting); methodology (supporting); supervision (supporting). **Radim Němeček:** Supervision (supporting); writing – original draft (supporting); writing – review and editing (supporting). **Jan Trna:** Conceptualization (supporting); project administration (supporting); writing – original draft (supporting); writing – review and editing (supporting). **Lumír Kunovský:** Project administration (equal); supervision (supporting); writing – review and editing (equal).

## FUNDING INFORMATION

This work was supported by the Specific University Research (grant no. MUNI/A/1224/2022).

## CONFLICT OF INTEREST STATEMENT

The authors declare that they have no conflict of interest.

## Data Availability

Data sharing is not applicable to this review article as no new data were created or analysed. The article, however, contains two tables and two schematic figures, which have been modified and created by the authors through reviewing the information, pooled from the already published literature/journal articles. All the references have been duly cited in this manuscript. No third‐party material (figures or tables) has been included in the current article.
